# Declining functional connectivity and changing hub locations in Alzheimer’s disease: an EEG study

**DOI:** 10.1186/s12883-015-0400-7

**Published:** 2015-08-20

**Authors:** Marjolein MA Engels, Cornelis J. Stam, Wiesje M. van der Flier, Philip Scheltens, Hanneke de Waal, Elisabeth CW van Straaten

**Affiliations:** Alzheimer Center and Department of Neurology, Neuroscience Campus Amsterdam, VU University Medical Center, Amsterdam, The Netherlands; Department of Clinical Neurophysiology and MEG center, Neuroscience Campus Amsterdam, VU University Medical Center, Amsterdam, The Netherlands; Department of Epidemiology and Biostatistics, Neuroscience Campus Amsterdam, VU University Medical Center, Amsterdam, The Netherlands; Nutricia Advanced Medical Nutrition, Nutricia Research, Utrecht, The Netherlands

## Abstract

**Background:**

EEG studies have shown that patients with Alzheimer’s disease (AD) have weaker functional connectivity than controls, especially in higher frequency bands. Furthermore, active regions seem more prone to AD pathology. How functional connectivity is affected in AD subgroups of disease severity and how network hubs (highly connected brain areas) change is not known. We compared AD patients with different disease severity and controls in terms of functional connections, hub strength and hub location.

**Methods:**

We studied routine 21-channel resting-state electroencephalography (EEG) of 318 AD patients (divided into tertiles based on disease severity: mild, moderate and severe AD) and 133 age-matched controls. Functional connectivity between EEG channels was estimated with the Phase Lag Index (PLI). From the PLI-based connectivity matrix, the minimum spanning tree (MST) was derived. For each node (EEG channel) in the MST, the betweenness centrality (BC) was computed, a measure to quantify the relative importance of a node within the network. Then we derived color-coded head plots based on BC values and calculated the center of mass (the exact middle had x and y values of 0). A shifting of the hub locations was defined as a shift of the center of mass on the y-axis across groups. Multivariate general linear models with PLI or BC values as dependent variables and the groups as continuous variables were used in the five conventional frequency bands.

**Results:**

We found that functional connectivity decreases with increasing disease severity in the alpha band. All, except for posterior, regions showed increasing BC values with increasing disease severity. The center of mass shifted from posterior to more anterior regions with increasing disease severity in the higher frequency bands, indicating a loss of relative functional importance of the posterior brain regions.

**Conclusions:**

In conclusion, we observed decreasing functional connectivity in the posterior regions, together with a shifted hub location from posterior to central regions with increasing AD severity. Relative hub strength decreases in posterior regions while other regions show a relative rise with increasing AD severity, which is in accordance with the activity-dependent degeneration theory. Our results indicate that hubs are disproportionally affected in AD.

**Electronic supplementary material:**

The online version of this article (doi:10.1186/s12883-015-0400-7) contains supplementary material, which is available to authorized users.

## Background

Alzheimer’s disease (AD) is a progressive neurodegenerative disease and a growing public health concern. At the cognitive level, AD is mainly characterized by memory impairment but it also affects other cognitive domains [1]. Meanwhile, AD patients show microscopic alterations in their brain, such as amyloid depositions and cell loss, which eventually may lead to macroscopic EEG changes. Cognition results from an optimal combination of local information processing and interregional integration of this information [2]. This communication can be macroscopically approximated by the measurement of functional connectivity using time series that reflect brain activity. A functional brain network can be constructed by taking all functional connections (i.e., edges of the network) between all regions (i.e., nodes of the network). In these networks, nodes that have a central position within the network and therefore are important to the network structure and integrity, are called hubs. Previous research has shown that the parietal brain region, including the precuneus and posterior cingulate gyrus, is an important hub region in the healthy brain [3]. In AD, this parietal hub region seems to be particularly affected [4]. Electroencephalography (EEG) measures electrical brain activity and is used to study functional connectivity and networks in AD. In a group of early-onset AD patients, we observed reduced hub status in the posterior- and occipital brain regions with EEG [5].

Studies of functional connections have revealed AD-related changes, in which functional connectivity is generally lower in AD [6, 7], specifically in the higher frequency bands [8, 9]. On the other hand, network characteristics seem to be altered in AD (e.g., [9–11]). It is however not known how the severity of the disease influences both functional connections and brain networks in AD.

In this EEG study, we studied the hub strength and location and evaluated functional connectivity as a function of disease severity. Furthermore, we subdivided the EEG electrodes into frontal, central and posterior regions. Our hypotheses are three-fold. Firstly, we hypothesize that functional connectivity is reduced in mild stages of the disease and decreases further with increasing disease severity. Secondly, we expect hub strength to decrease in the same areas as the functional connectivity disruptions. Lastly, we expect hub strength to decrease (most likely in regions with decreasing functional connectivity) and therefore, we expect that other regions will become relatively more hub-like (a shifted hub location).

## Methods

### Subjects

We included 318 patients with probable AD and 133 non-demented controls with available EEG [12, 13]. The AD group was classified into tertiles based on Mini Mental State Examination (MMSE) (mild, moderate and severe, Table 1). The participants are a subset of the Amsterdam Dementia Cohort [14]. Standardized dementia screening included patient history (including an informant based history of the patient, if available), neurological and cognitive examination, EEG, magnetic resonance imaging (MRI) of the brain, and laboratory tests. Patients were diagnosed with probable AD according to the National Institute of Neurological and Communicative Disorders and Stroke and the Alzheimer’s Disease and Related Disorders Association (NINCDS-ADRDA) criteria during a multidisciplinary consensus meeting [1, 15]. The non-demented control group consisted of age-matched patients with subjective cognitive complains but without abnormalities on formal testing (i.e., criteria for mild cognitive impairment or any psychiatric disorder were not fulfilled). All participants gave written informed consent for the use of their clinical data for research purposes [14]. The ethical review board of the VU University Medical Center approved this procedure.

**Table 1:** Subject characteristics

	Controls	AD patients		
		Severe	Moderate	Mild
N	133	117	96	105
Age, Years^a^	67.8(6.3)	68.1(8.5)	70.7(8.8)	69.9(9.6)
Sex, Female	59(63 %)^b^	62(53 %)	52(54 %)	40(38 %)
MMSE-score^a^	28.5(1.3)^c^	15.4(3.5)	21.5(1.1)	25.8(1.6)
Disease duration, years^a^	n.a.	4.1(2.5)	3.5(2.1)	3.2(1.9)
Using AChEI^a^	1(0.8 %)	8(6.8 %)	5(5.2 %)	5(4.8 %)
Education^a d^	5.4 (1.2)	4.2 (1.5)	4.8 (1.3)	5.3 (1.2)

The data is represented as mean (SD) or number (percentage)

*AChEI* Acetyl-cholinesterase inhibitor, *MMSE* mini mental-state examination

^a^These variables where tested using ANOVA. We corrected the results for multiple comparison using a post-hoc Bonferroni test

^b^gender differences of *p* < 0.01 were found between mild AD and severe/moderate AD

^c^MMSE differences of p < 0.01 were found between all group combinations

^d^Level of education was rated according to Verhage [51]

### EEG recording

Details about EEG recordings and technical aspects have been previously described [5, 12, 13]. In short, we recorded EEG with 21 electrodes at the positions of the 10–20 system with a sample frequency of 500 Hz and the electrode impedance of below 5kΩ (BrainLab, OSG b.v., Rumst, Belgium). Initial filter settings were: time constant 1 s; low pass filter at 70 Hz. Patients sat with eyes closed in a slightly reclined chair in a sound attenuated room. EEG technicians were alert on keeping the participants awake. Four epochs of 4096 samples were found to represent a stable EEG measure in a subset of our dataset (see Additional file 1). Therefore, we selected four artifact free epochs (containing as little as possible eye blinks, slow eye-movements, excess muscle activity, ECG artifacts, etc.) of 4096 samples and a common average reference were selected from each EEG (by HdW). EEG channels were clustered into three different regions to perform regional analysis: anterior (Fp1, Fp2, F7, F3, Fz, F4, F8), central (T3, C3, Cz, C4, T4), and posterior (T5, P3, Pz, P4, T6, O1, O2).

### Functional connectivity

Functional connectivity was assessed using the Phase Lag Index (PLI), which is a measure of statistical interdependencies between time series based upon the asymmetry in the distribution of instantaneous phase differences [16]. The PLI ranges between zero and one in which zero indicates no coupling strength and one refers to maximal coupling strength. The PLI is less sensitive to volume conduction than most other frequently used measures for functional connectivity [17]. BrainWave software version 0.9.125 (available at http://home.kpn.nl/stam7883/brainwave.html) was used to calculate PLI in five frequency bands (delta band 0.5–4 Hz, theta band 4–8 Hz, lower alpha band 8–10 Hz, upper alpha band 10–13 Hz, beta band 13–30 Hz). We did not take into account the gamma band (>30 Hz) because this fast activity cannot reliably be distinguished from muscle artefacts [18]. In every subject, the mean PLI was calculated by taking the mean of four epochs. In addition to the mean whole-brain PLI, we computed regional PLI by averaging the PLI values of the electrodes in the anterior, central and posterior clusters.

### Hub status

From the adjacency matrix containing the PLI values, we constructed the minimum spanning tree (MST), which is a unique subgraph that connects all nodes (EEG electrodes in our study) of a network by the strongest connections (defined as the links with the highest PLI values) without forming cycles (i.e., loopless) [2] using Kruskal’s algorithm [19]. From the MST and for each node, its importance in the functional network was established with the betweenness centrality (BC). This is a measure of the hub-status and is defined as the number of paths between node-pairs that run through a specific node, divided by the total number of paths from any node to all other nodes in the MST. A network node with a relatively high BC-value compared to other network nodes is suggestive for a hub-region in that network.

### Hub visualization plots

Color-coded BC values of the MST per electrode were plotted on a 2-D head model using biharmonical spline interpolation in MatLab® 2012b (The mathworks, Massachussets, USA) [20]. In addition, using the MatLab dot product function, we calculated a vector based upon BC values of the MST over both the x- and y-axis (taken the 10–20 system channel locations into account). The acquired x- and y-vectors were then plotted onto the head model to visualize the “center of mass” of the BC. This vector is based upon the BC values of all nodes and is located on the spot where the surrounding BC values are balanced (for example: if the BC values of all network nodes are equal, the center of mass vector will be located exactly in the middle of the brain while if one node in the network has a higher BC value, the center of mass vector will shift towards that specific node). Hereby, the hub status of the three patient groups (mild, moderate and severe AD) and the control group can be displayed both visually (i.e., both the color-coding of the BC values and the displayed center of mass vector on a head model) and quantitatively (i.e., y-value of the mass center). The y- values, representing the front-to-back direction, were used for statistical analyses. Note that an x- and y value of 0 represent the exact middle of the graph (c.q. EEG electrode Cz).

### Statistical analysis

IBM SPSS Statistics version 20 for Mac was used for statistical analyses. Differences between baseline group characteristics were tested with *χ*^2^-tests and one-way ANOVA with post-hoc Bonferroni tests. Natural log transformation [y = ln(x)] was applied on PLI and BC values to obtain normal distributions of these measures with an addition of 1•10^−24^ to avoid zeros in the data. To test PLI and BC group differences, we used multivariate general linear models in four regions (anterior, central, posterior and global) and five frequency bands (delta, theta, lower alpha, upper alpha and beta). We tested the influence of the severity of the disease (entered as continuous independent variable: controls – mild – moderate- severe AD) in the multivariate general linear model with gender as covariate in order to obtain the p for trend (note that this p for trend represents a significant gradual change over the groups). The multivariate general linear models were conducted in 3 sessions: (1) log-transformed PLI values as dependent variables with the group as continuous independent variable; (2) log-transformed BC values as dependent variables with the group as continuous independent variable; (3) x- and y values of the center of mass (as described in the previous paragraph) as dependent variables with the groups as continuous independent variables. We used a Bonferroni correction in order to correct for the number of groups. Statistical significance was set at *p* < 0.05 for PLI and BC values and *p* < 0.01 for subject characteristics. A two-tailed Spearman correlation analysis was performed across and within the AD groups with MMSE-scores. Statistical significance was corrected for the number of tests by dividing the preferred *p*-value (*P* < 0.05) by the number of tests.

## Results

### Subject characteristics

Subject characteristics of the four groups (controls, mild AD, moderate AD and severe AD) are presented in Table 1. Mean age did not differ between groups. The mild AD group contained more females than the severe AD and moderate AD groups. There was no difference in education and the use of cholinesterase inhibitors between groups. The estimated disease duration was not different within the three AD groups. We did not find differences between the amplitudes of the EEG signals in any of the regions between groups.

### Functional connectivity

We found an association between increasing disease severity and decreasing PLI in the lower alpha band in the posterior region (PLI-values: controls 0.274 ± 0.107, mild AD 0.249 ± 0.091, moderate AD 0.236 ± 0.083, severe AD 0.238 ± 0.095; p for trend = 0.03). This indicates that functional connectivity reduction is associated with increasing disease severity. Other regions and bands did not show any associations with disease severity.

### Hub location and strength

**Fig. 1:**
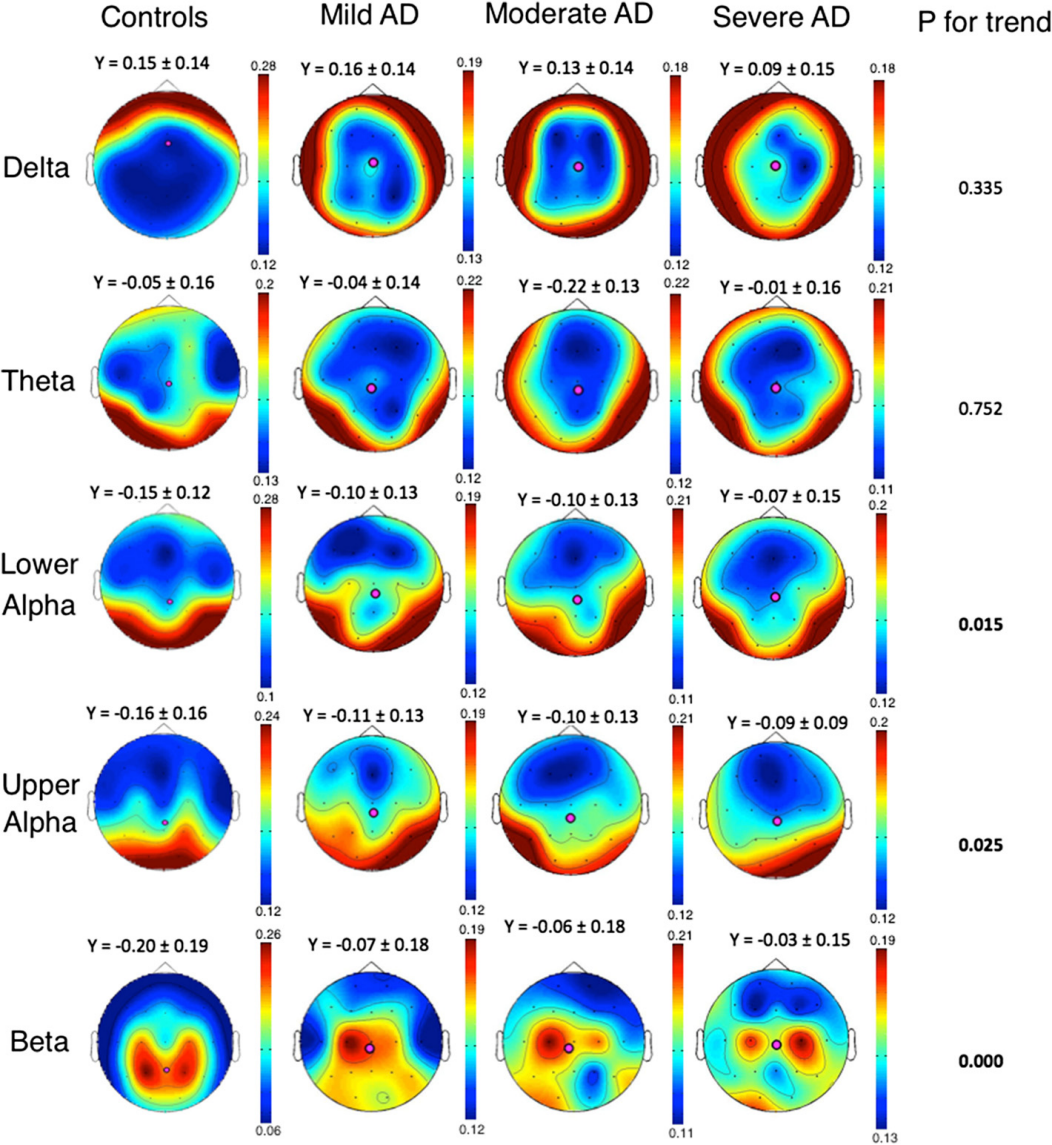
Betweenness centrality values of the minimum spanning tree. Betweenness centrality values plotted on a 2-D head model for different groups and frequency bands. y-values represented the location of the mass center on the y-axis in which zero represent the exact center of the head plot ± standard deviation. The p for trend value represents the *p*-value of the multivariate general linear model with Bonferroni post-hoc analysis

The BC values of the nodes in the MST, as an indication of the node importance, are shown in Table 2 (raw values, p for trend). We found increasing BC values in the lower alpha band global and in the anterior region; in the upper alpha band global, in anterior and central regions; and in the beta band in the anterior region. We observed decreasing BC values in the beta band in the posterior region. Figure 1 presents head plots of BC values with the center of mass marked in all frequency bands and groups to visualize the changing location of the center of mass of the BC values. In the delta and theta bands, the center of mass of the controls is located in the anterior and central regions respectively while in the alpha bands and beta bands it is located in the posterior regions. The y-values of the center of mass of the AD patients are located centrally in all frequency bands. The back-to-front shifting of the center of mass from the posterior to the central regions, as indicated by the y-values, increased with increasing disease severity in the alpha and beta bands (p for trend = 0.011 in alpha1; p for trend = 0.025 in alpha2; and p for trend < 0.000 in beta). Generally, in the higher frequency bands the most important nodes (as indicated by a high BC value) were located in the posterior brain regions in controls and, with increasing disease severity, were becoming relatively less important. The left-to-right shifting of the center of mass was significant only in the beta band (p for trend = 0.012) indicating a shift to the right side of the brain in the most severely affected AD patients.

**Table 2:** Minimum spanning tree-based betweenness centrality differences between groups

Frequency band	Region	Control	Severe AD	Moderate AD	Mild AD	P for trend
		Mean BC (SD)	Mean BC (SD)	Mean BC (SD)	Mean BC (SD)	
Delta	global	0.154 (0.014)	0.154 (0.013)	0.155 (0.012)	0.154 (0.014)	N.S.
	anterior	0.206 (0.043)	0.188 (0.050)	0.199 (0.045)	0.209 (0.047)	N.S.
	central	0.120 (0.045)	0.122 (0.054)	0.129 (0.047)	0.121 (0.046)	N.S.
	posterior	0.126 (0.039)	0.144 (0.043)	0.130 (0.039)	0.123 (0.043)	N.S.
Theta	global	0.158 (0.028)	0.157 (0.013)	0.157 (0.016)	0.157 (0.014)	N.S.
	anterior	0.153 (0.057)	0.157 (0.053)	0.154 (0.049)	0.155 (0.054)	N.S.
	central	0.142 (0.049)	0.149 (0.046)	0.142 (0.047)	0.142 (0.043)	N.S.
	posterior	0.174 (0.044)	0.162 (0.049)	0.171 (0.040)	0.170 (0.044)	N.S.
Lower alpha	global	0.154 (0.015)	0.159 (0.014)	0.156 (0.012)	0.158 (0.013)	**0.02**
	anterior	0.120 (0.039)	0.144 (0.045)	0.134 (0.044)	0.136 (0.045)	**0.04**
	central	0.133 (0.048)	0.145 (0.049)	0.142 (0.047)	0.142 (0.044)	N.S.
	posterior	0.202 (0.039)	0.184 (0.046)	0.189 (0.043)	0.193 (0.038)	N.S.
Upper alpha	global	0.156 (0.013)	0.161 (0.012)	0.160 (0.014)	0.161 (0.014)	**0.01**
	anterior	0.118 (0.052)	0.140 (0.046)	0.133 (0.044)	0.129 (0.041)	**0.01**
	central	0.150 (0.056)	0.157 (0.054)	0.160 (0.049)	0.163 (0.053)	**0.02**
	posterior	0.200 (0.045)	0.186 (0.042)	0.188 (0.042)	0.192 (0.040)	N.S.
Beta	global	0.142 (0.015)	0.142 (0.017)	0.146 (0.018)	0.145 (0.016)	N.S.
	anterior	0.082 (0.053)	0.127 (0.055)	0.120 (0.060)	0.117 (0.058)	**0.00**
	central	0.185 (0.059)	0.170 (0.066)	0.178 (0.069)	0.185 (0.061)	N.S.
	posterior	0.172 (0.054)	0.138 (0.058)	0.148 (0.054)	0.143 (0.057)	**0.03**

The data are presented as raw minimum spanning tree-based betweenness centrality (BC). Note that raw data are presented, while analyses were performed on log-transformed data. Significance was obtained by a multivariate general linear model and Bonferroni post-hoc analysis. Significant differences between the patient groups and the control group are printed in bold. *AD* Alzheimer’s disease, *BC* betweenness centrality, *SD* standard deviation

### Correlation with MMSE scores

**Fig. 2:**
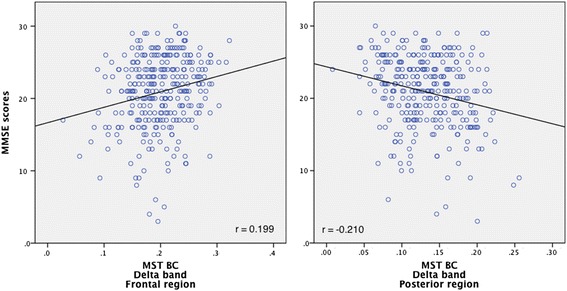
Correlation plots with cognition. This figure represents Spearman’s correlations between MMSE-scores and the delta band in the frontal region (*left panel*) and posterior region (*right panel*)

Since we measure 5 frequency bands, 4 regions and 3 AD groups, we set the *p*-value threshold for significance to 0.00042. We found no significant correlations with MMSE. Next, we merged the 3 AD groups into 1 group containing all AD patients. For this analysis, we set the *p*-value threshold for significance to 0.00125. We found a positive significant correlation with MMSE in the BC-values in frontal region in the delta band (Spearman’s *r* = 0.199; *p* < 0.001) and a negative correlation with MMSE in the BC-values in the posterior region in the delta band (Spearman’s *r* = −0.210; *p* < 0.001), as represented in Fig. 2.

## Discussion

In this study on topological patterns of physiological brain activity, we found that a decrease in the functional connectivity in the posterior brain regions was associated with increasing disease severity in the lower alpha band. In addition, the locations of the hubs in the functional networks of AD patients were located towards anterior brain regions compared to the hubs of the control networks, with a significant shift to a more anterior location in the more severely affected patients. The relative node importance of the frontally and centrally located brain areas, as quantified with the BC of the MST, increased with disease severity in AD.

Lower functional connectivity in AD has previously been reported in studies using different modalities, but the pattern of this functional connectivity and the methodology varied considerably between studies. In studies with high temporal resolution time series (EEG and magnetoencephalography (MEG)), functional connectivity in AD was found to be decreased in the higher frequency bands (alpha and beta) [9, 21–26] as well as the lower frequency bands (theta) [22, 23] and involving mainly brain regions that are connected by long (corticocortical) fibers [24, 27]. In addition, the location of the largest decrease in patients compared to controls varied over the studies: main differences between groups were found in the anterior and central regions [18, 28, 29] as well as the posterior regions [21] and in one study, both regional increases and decreases were found [30]. Functional connectivity increases of slower oscillations (theta band) were also reported [8, 31]. In studies with lower temporal but high spatial resolution (functional magnetic resonance imaging (fMRI) and positron emission tomography (PET)), a similar pattern has been found with regionally dependent increases as well as decreases in functional connectivity but with a tendency for a general decrease in AD (for a review see: [32]). These results indicate that the interpretation of functional connectivity changes in AD is, at least to some extent, dependent on the method used for the analysis. In addition, it can be conceived that during the course of the disease the functional connectivity is fluctuating, with a possible initial increase [33] and a later decrease. Therefore, differences in inclusion criteria of AD patients across studies could partly account for the differences in the results [4]. We included patient groups of different disease severity and studied the gradual effect of the AD severity on functional connectivity. Our results indicate that AD severity correlates with a functional connectivity decrease in the posterior brain areas in the lower alpha band. These results give rise to the hypothesis that loss of functional networks might be more valuable than increasing amyloid burden, which is supposed to have plateau’d at the stage of dementia [34].

The posterior brain areas are main hub regions, and are known to be involved in AD [35]. In healthy subjects, the posterior brain areas, including the precuneus and the posterior cingulate gyrus, contain hubs with many functional connections to other brain areas [36–38] and are important for intellectual performance [39]. Also, hubs seem electrically more active, as shown in an EEG simulation study [4, 40]. Meanwhile, these hubs are more likely to be abnormal in a brain disorder like AD [41]. Previously, the amyloid depositions were found to have a predilection for high activity brain areas [42]. In addition, glucose metabolism in AD showed reduced activity in the cortex of the posterior cingulate gyrus [43, 44] and precuneus [45, 46]. We reported a shifted hub region, from posterior in controls, to more central regions in AD patients. However, since EEG has a low spatial resolution, any assumptions about regional effects should be made with caution. The functional meaning of the relocation of hubs to more anterior regions (i.e., EEG sensors) might have it’s origin in the heterochronicity of the pathophysiological processes in AD. This means that the pathological pattern is different in patients early in the disease as compared to later stages of the disease. This causes that the gradually degrading posterior region with rising disease pathology in this region to eventually be incapable of effectively conducting electrical activity.

Patients were diagnosed with AD based on clinical criteria using a standardized diagnostic protocol and international criteria [1, 15]. The control subjects presented at the clinic with subjective memory complaints and can therefore not strictly be considered healthy. However, this group is clinically relevant since they represent daily practice in the memory clinic. The choice for the functional connectivity measure influences the results. In this study, we used the PLI. This measure might be biased towards long distance connectivity, because all zero-lag (mostly short distance) connections are discarded. However, the main advantage of this approach is the reduction of bias due to volume conduction and activity from common sources [16]. Thus, the PLI may be an underestimation of functional connectivity and therefore, in our study of a large cohort, would show an underestimation of the real disease-effect.

The number of epochs used for analyses may influence the PLI results. In our analyses, we used 4 epochs of 8.192 s (4096 samples). We found that 4 epochs give as reliable PLI values as 5,6,7 or 8 epochs (see Additional file 1).

When comparing networks, several choices have to be made to handle networks of different sizes (number of nodes) and connection strengths. These choices influence the results of the network analysis and are arbitrary [47]. The MST has the advantage of giving a unique representation of a connectivity matrix since no arbitrary choices have to be made. It is the minimal connected sub-network consisting of the strongest connections without forming cycles. Therefore, the MST can be considered as a backbone of the network that likely includes most of the important connections in the network [28, 48].

Different centrality indices result in different values for the same graph. We choose the betweenness centrality as a measure for centrality in brain networks. It has previously been proposed to be robust to measure centrality of nodes in networks [49]. Another often-applied centrality measure is the node degree that indicates the number of connection of a node in the network. Although the number of shortest paths through a node and the number of connections of that node are likely to be related, node degree is not sensitive to so-called connector hubs [50]. Connector hubs are thought to connect high degree hubs to each other and therefore have a relatively low degree but at the same time include many shortest paths (e.g., a high betweenness centrality).

## Conclusions

In conclusion, we observed that functional connectivity in AD decreases in the posterior brain regions in the lower alpha band in a disease severity dependent fashion. Second, we described a more widespread disease severity related relative increase in anterior hub strength compared to the posterior brain areas. Third, we found that the hub location shifts gradually from posterior regions in controls, towards more central regions in AD. All findings were specific for the higher frequency ranges (lower alpha, upper alpha and beta bands). Changes in hub status were more outspoken than the functional connectivity changes, which suggest that hubs are disproportionally affected in AD.

## Additional file

Additional file 1:
**The influence of the number of epochs.** The number of epochs used for analyses influences the PLI outcomes. This supplement, including 1 figureand 1 table show that the PLI values become stable after 4 epochs of 8.192 seconds (4096 samples). (ZIP 66 kb)
