# Supplementary material for: Declining functional connectivity and changing hub locations in Alzheimer’s disease: an EEG study
Source: BMC Neurol. 2015 Aug 20;15:145. doi: 10.1186/s12883-015-0400-7 (PMC4545875; doi:10.1186/s12883-015-0400-7)
Supplement: Additional file 1: — The influence of the number of epochs. The number of epochs used for analyses influences the PLI outcomes. This supplement, including 1 figureand 1 table show that the PLI values become stable after 4 epochs of 8.192 seconds (4096 samples). (ZIP 66 kb) [file 12883_2015_400_MOESM1_ESM.zip › additional file 1/1520768448163642_add1.docx]

**Additional file 1**

*Number of epochs*

We selected artifact free epochs (i.e. we carefully discarded epochs that showed signs of drowsiness, muscle artifacts, eye blinks or eye movements) of 4096 samples (8.192 seconds) for a random subset of our data. The number of subjects used for this subset was √N = √451 = 21 subjects. The maximum number of artifact free epochs for every subject ranged from 5 to 19 (mean 12; standard deviation 3.5). We calculated the PLI values over all artifact free epochs and repeated the measurement after deleting 1 epoch until only 1 epoch was left. In this way, information was obtained about the variability of PLI assessment as a function of number of epochs used in the averaging procedure.

First, we set the PLI values of 1 epoch for every subject at a fixed value (100). Next, we calculate the PLI values of (averages over ) 2, 3, 4, …, n epochs relative to the fixed value by multiplying by 100 and dividing by the PLI value of the reference epoch. We repeated this procedure for all epochs.

Thereafter, we successively calculated the mean and the standard deviation of the epoch numbers larger than the epoch number that has been set to 100, where the number of epochs decreases with every next step and we assume that the more epochs, the more stable the data will be (Figure S1). Figure S1 shows an increase in stability of the PLI values with increasing number of epochs. The red line indicates the number of subjects enrolled in the calculation. Since the maximum number of epochs we could select differed across subjects, the red line is declining. Independent t-statistics showed differences between the PLI values (based upon the fixed values) between 4 epochs and 1,2 and 3 epochs, while we did not find differences between 4 epochs and 5,6,7 and 8 epochs. Therefore, 4 epochs give as reliable PLI values as 5,6,7 or 8 epochs. From 9 epochs on, the variance decreases while also the number of subject enrolled decreases (shown in Table S1).

**Figure S1 – Stability of PLI values with increasing number of epochs.**

Red line indicates the number of subjects enrolled in the calculation. PLI values have been calculated relatively against a fixed value (100) for every number of epochs, as described in the supplementary materials.

**Table S1 – p values of independent t-tests.**

| 4 epochs compared with: | p |
| --- | --- |
| 1 epoch | **0.000** |
| 2 epochs | **0.000** |
| 3 epochs | **0.000** |
| 5 epochs | 0.131 |
| 6 epochs | 0.155 |
| 7 epochs | 0.066 |
| 8 epochs | 0.146 |
| 9 epochs | **0.021** |
| 10 epochs | **0.000** |
| 11 epochs | **0.000** |
| 12 epochs | **0.001** |
| 13 epochs | **0.001** |
| 14 epochs | **0.006** |
| 15 epochs | 0.052 |
| 16 epochs | 0.088 |
| 17 epochs | 0.027 |
|  |  |

Results of independent t-tests between the relative PLI values of the different numbers of epochs. Bold p-values are significantly different.
