# Supplementary figures and images for: Declining functional connectivity and changing hub locations in Alzheimer’s disease: an EEG study
Source: BMC Neurol. 2015 Aug 20;15:145. doi: 10.1186/s12883-015-0400-7 (PMC4545875; doi:10.1186/s12883-015-0400-7)

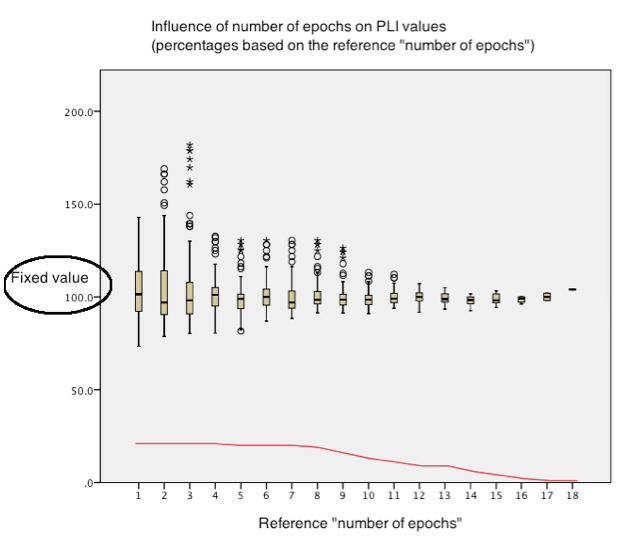

Supplement: Additional file 1: — The influence of the number of epochs. The number of epochs used for analyses influences the PLI outcomes. This supplement, including 1 figureand 1 table show that the PLI values become stable after 4 epochs of 8.192 seconds (4096 samples). (ZIP 66 kb) [file 12883_2015_400_MOESM1_ESM.zip › additional file 1/1520768448163642_add2.png]
